# Efficacy of Pumpkin Ointment in Treatment of Chronic Hand Eczema: A Randomized, Active-Controlled, Double Blind Clinical Trial

**DOI:** 10.18502/ijph.v49i7.3588

**Published:** 2020-07

**Authors:** Alemeh KHADEMI, Parvin MANSURI, Daryoush PAHLEVAN, Mahbubeh BOZORGI, Malihe NASIRI, Somayeh HEJAZI, Zahra AZIZIAN, Laila SHIRBEIGI

**Affiliations:** 1. Department of Traditional Medicine, School of Persian Medicine, Tehran University of Medical Sciences, Tehran, Iran; 2. Skin and Stem Cell Research Center, Tehran University of Medical Sciences, Tehran, Iran; 3. Research Center for Social Determinants of Health, Semnan University of Medical Sciences, Semnan, Iran; 4. Department of Traditional Pharmacy, School of Iranian Traditional Medicine, Tehran University of Medical Sciences, Tehran, Iran; 5. Department of Biostatistics, School of Paramedical, Shahid Beheshti University of Medical Sciences, Tehran, Iran; 6. Department of Dermatology, Iran University of Medical Sciences, Tehran, Iran

**Keywords:** Hand eczema, Traditional medicine, Persian, Pumpkin, Almond, Betamethasone

## Abstract

**Background:**

Hand Eczema (HE) is chronic skin disease with a high prevalence in population. It has negative impact on the quality of life. Due to the public interest in herbal remedies, we attempt to assess the efficacy of pumpkin ointment in treatment of chronic HE in this research.

**Methods:**

This study was conducted in an outpatient clinic at Imam-Khomeini Hospital in Tehran (Iran) from May 2015 to Nov 2016. We performed a double-blind trial on 60 patients with chronic HE randomized to four groups included pumpkin, betamethasone, eucerin and almond ointment (n=15 for each group) for 28 days. Patients were ordered to apply ointments twice a day. Hand Eczema Severity Index (HECSI) and Dermatology Life Quality Index (DLQI) of the patients were evaluated by a dermatologist on the 1st, 14th and 28th d after the start of treatment.

**Results:**

Patients’ DLQI scores in pumpkin and betamethasone group was significant and pumpkin group showed a better response in quality of life (*P*=0.001). Betamethasone and pumpkin ointment were effective and showed significant improvement compared with almond and eucerin and reduce HECSI scores (*P*=0.002 and *P*=0.012 respectively). Betamethasone ointment outcome on HECSI scores in comparison with topical pumpkin was significant (*P*<0.001). No clinically adverse effects were observed.

**Conclusion:**

Although pumpkin ointment showed a better response in patients’ DLQI in HE but it was less effective than betamethasone in decreasing HECSI.

## Introduction

Chronic hand eczema (HE) is the most disabling dermatological complaint, affects hands with poor prognosis and leads to significant financial, social, and emotional costs for patients and society (1, 2). Annual prevalence of Chronic HE has been reported 10% to 14 % (2, 3). Due to high prevalence of HE and its chronic course, it has significant impairment in patient’s quality of life. It prevents daily activities, causes psychosocial impairment and leads to sleep disorders, anxiety and depression (2,4). Erythema, papules, vesicles, cracks (fissures), pain, scaling, hyperkeratosis, as well as pruritus are typical clinical signs of eczema (5).

HE has multiple causes that are irritant contact dermatitis, atopic dermatitis, and allergic contact dermatitis (6). Topical corticosteroids are the first line of treatment. Moreover, patients are treated with broad variety strategies, which include lifestyle modifications, oral antihistamines and short course of systemic corticosteroids (7). On the other hand, there are increasing interests in Traditional, Complementary and Alternative Medicine (TCAM), especially by patients with chronic illnesses (8, 9). Therefore, there is a place for assessment of CAM modalities such as herbal medicine for these patients (10, 11). *Cucurbita moschata* flesh and seeds are one of the plants highly regarded in traditional Persian medicine to reduce skin damage and acts as an anti-inflammatory agent. Furthermore pumpkin pulp has chemical composition such as β-carotene, fatty acids, moisture and flavonoids and has proven that it can be used for many skin disorders such as dermatitis orally or topically (12, 13). However, there was no clinical experiment to show the effect of pumpkin extract on HE.

This study aimed to evaluate the efficacy of pumpkin ointment in patients with mild to moderate HE.

## Materials and Methods

This was a 4-week, clinical trial which had 4 parallel groups. We evaluated the effectiveness of topical pumpkin ointment of *C. moschata* oil-extract by voucher number of 6759-TEH and compared it with topical 0.1% betamethasone ointment (from Emad Darman Pars co), almond ointment (from Barij Essence co) and eucerin (from Emad Darman Pars co) on patients with HE. All subjects were asked to sign an informed consent before participating in the study. This study conducted in an outpatient clinic at Imam-Khomeini Hospital in Tehran (Iran) from May 2015 to Nov 2016.

The study was licensed by the Ethics Committee of the National Research Centre of Medical Sciences of Tehran, Iran. Consent forms were obtained from all the participants. The trial registered code is IRCT2014012916412N1.

The study was designed as a randomized, double-blind study. Sixty male or female patients (age, 18–60 yr) who had chronic HE and did not use topical medication for at least 2 wk or systemic medication for one month before trial was included. Other Inclusion criteria were lack of pregnancy and lactation, lack of a history of contact dermatitis to prescription drugs, lack of local infection, lack of obsessive-compulsive disease concerned with over-washing and lack of any autoimmune system diseases. Patients that had skin allergy during treatment, lack of cooperation and erratic consumption of medicines, impossibility of keeping track of patient; need to receive medicines that interfere with treatment withdrawal of treatment for any reason and evidence of active infection at their lesions were excluded from the study.

At first, the patients were randomly divided into four parallel groups by using a block-randomization list prepared by the project bio-statistician (A, B, C, and D groups). After confirming the diagnosis of chronic HE by two dermatologists, participants were enrolled in the study.

By using the randomization list the drugs were prepared in same form and color tubes and tagged A, B, C, and D and numbered for each patient by the study’s pharmacist that did not have any role in the intervention. Drug delivery to the patient was done by the traditional Persian medical resident who was blind about drugs coding. Throughout the study, the patients were blinded too.

At the end of the study, the patient’s information was analyzed by a dermatologist that was blind about grouping and drug coding. The patients were randomly allocated to four groups include pumpkin, almond, betamethasone and eucerin ointment sequentially. The project biostatistician assigned the patients to four groups based on randomization function in excel software by using a block-randomization list. There are fifteen patients in each group (12).

The patients were instructed to apply maximally one gram of ointments (estimated by Finger Tip Unit (FTU) twice a day for 4 wk (28 d) topically on eczematous lesions. All patients were trained to wash their hands with glycerin soap and moisturize them with eucerin ointment. During the study, they should not use any other topical medication at the site of the lesions. Additionally, all of the patients were advised to avoid repeated hand washing and any other eczema predisposing factors (15).

### 

#### Pumpkin oil preparation

Fleshy fruit parts of *C. moschata* was purchased during Jan 2015 from a local herbal market, Tehran, Iran. The taxonomic identification of the plants was confirmed by the botanist of the Department of Cultivation and Development of Pharmacognosy, Faculty of Pharmacy, Tehran University of Medical Sciences by voucher number of 6759-TEH. Pumpkin oil was prepared according to mentioned methods in traditional Persian medicine manuscripts (16). Firstly, 300 g. of crushed pumpkin fruit was macerated in 500 mL water for 48 h to preparing aqueous extract. Then, 200 mL of prepared extract was added to equal amount of almond oil and this mixture was heated gently up to complete water evaporation. Pumpkin oil was gradually added to eucerin and mixed until a homogeneous ointment was obtained. The ratio of oil to eucerin was 30% which was the highest amount used.

### Standardization of pumpkin oil

Fatty acid profiles of pumpkin oil were determined using Gas Chromatography (GC). Gas chromatograph (Shimadzu, GC-2010, jpan) equipped with a flame ionization detector (FID) and capillary column (60m×0.25mm i.d.; film thickness 0.20μm). The GC column temperature gradient was adjusted to 90 °C for 7 min, increased up to 240 °C with a rate of 5 °C /min. Then, the temperature was held at 240 °C for 20 minutes. Temperature of both injector and detector was 260 °C. Oleic (50%) and linoleic acids (20%) were determined as the main fatty acid composition. The stability of the ointment was assessed for 3 months. Ointments were freshly prepared for each month during the study, so no preservative was added to the product. In this clinical trial, final formulation for microbiological tests was controlled based on USP XXIV (U.S. Pharmacopoeia 24-NF 19)(17).

### Statistical analyses

Demographic data and clinical characteristics of the patients in all four groups were compared. Statistical analysis was done by ANOVA and Fisher Exact test and repeated measures ANOVA. The statistical analysis that we applied were SPSS, ver. 21.0 (Chicago, IL, USA). All statistical comparisons were based on a significant level of *P*<0.05.

## Results

**Fig. 1:**
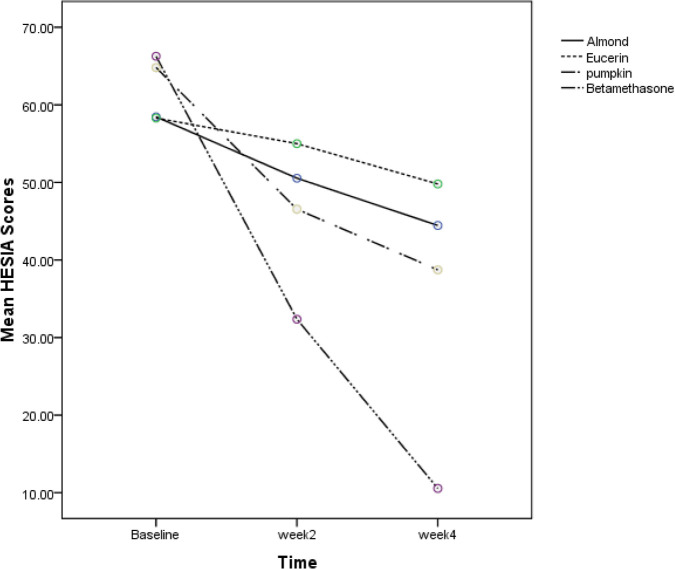
The comparison of HECSI scores in four groups at baseline, week 2 and week 4 of therapy

**Fig. 2:**
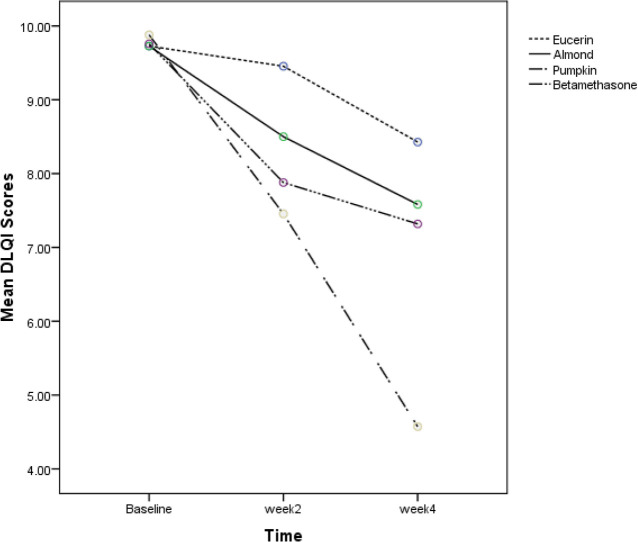
The comparison of DLQI scores in four groups at baseline, week 2 and week 4 of therapy

Seventy-seven patients were assessed for the study. Sixty patients were enrolled in the study in four groups. Fifteen patients in each group . Characteristics of participants at baseline are demonstrated in Table 1. HECSI and DLQI scores measured at the beginning of the study in four groups had no significant difference. (*P*=0.58 and *P*=0.98, respectively). Changes in HECSI and DLQI scores during the study are displayed in Figs. 1 and 2. There were significant differences in the score of HECSI between betamethasone and pumpkin groups (*P*<0.002). Pumpkin group had significant reduction in DLQI scores compared with patients used beta-methasone, eucerin and almond ointment (*P*<0.001). In Tables 2 and 3 Mean HECSI and DLQI scores during the treatment in four groups has been shown respectively. No side effects were observed during the study in all groups.

**Table 1:** Baseline characteristics of patient with chronic hand eczema

*** Characteristics ***	*** Eucerin (n = 13) ***	*** Almond (n = 13) ***	*** Betamethasone (n = 13) ***	*** Pumpkin (n=14) ***	*** P-value ***
Age, years (Mean ± SD)	36.18±6.95	37.27±7.62	36.55±5.11	37.91±5.05	0.75
Gender, no. (%) male	10(76.9)	11(84.6)	10(71.4)	10(83.3)	0.78
Medical history, no. (%)					
Asthma	1(7.70)	1(7.70)	1(7.14)	0(0)	0.45
Allergic rhinitis	4(30.77)	3(23.08)	5(35.71)	3(25)	
Atopic dermatitis	3(23.08)	2(15.38)	3(21.43)	2(16.67)	
Psoriasis	1(7.7)	1(7.7)	0(0)	0(0)	
Occupational exposures	4(30.77)	4(30.77)	3(21.43)	3(25)	
Positive family history of atopy	5(38)	5(38)	6(43)	4(33)	
Duration of disease, years					
Median (range)	1(0.5–18)	1.5(1–20)	1.8(0.9–22)	1.6(1–24)	0.68
HECSI ^†^ (at baseline)					
Mean ± SD	58.30±09.69	58.45±13.39	66.27±26.29	64.82±12.72	0.58
Median (range)	51(40–79)	55.5(46–76)	66(23–99)	66(32–79)	
DLQI ^‡^ (at baseline)					
Mean ± SD	9.73±0.79	9.73±0.74	9.76±1.67	9.88±0.62	0.98
Median (range)	9(8–11)	9(8–11)	10(7–11)	10(9–11)	

† HECSI: Hand Eczema Severity index. Score ranges 0 to 360, (0: no hand eczema, 360: severe disease).

‡ DLQI: Dermatology life Quality Index. Score ranges 0 to 30, The higher the score, the lower the quality of life. DLQI Scores 0–1= no effect; 2–5 = small; 6–10 = moderate; 11–20 = very large; 21–30 = Severe impact on patient’s life

**Table 2:** Changes in HECSI^*^ scores, during the course of treatment (at baseline, week 2 and week 4 by treatment groups)

*** Groups ***	*** Baseline ***	*** After 2 Wk ***	*** After 4 Wk ***
Eucerin			
Mean ± SD	58.30±09.69	55.00±06.30	49.80±3.11
Median (range)	51(40–79)	50(34–70)	40(38–60)
Almond			
Mean ± SD	58.45±13.39	50.36±12.17	44.09±8.20
Median (range)	55.5(46–76)	55(46–69)	49(46–57)
Pumpkin			
Mean ± SD	64.82±12.72	46.54±8.80	38.72±4.51
Median (range)	66(32–79)	46(28–62)	40(32–45)
Betamethasone			
Mean ± SD	66.27±26.29	32.36±18.67	10.54±9.05
Median (range)	66(23–99)	31(11–67)	8(5–26)
* P * -value	0.58	0.002	0.002

* HECSI: Hand Eczema Severity index

HECSI scores that measured at the beginning of the study in four groups had not significant difference. (*P*=0.58)

HECSI scores that measured in the day 14th and 28th day of the study in four groups had significant difference (*P*<0.002)

**Table 3:** Changes in Mean DLQI^*^ scores, during the course of treatment (at baseline, week 2 and week 4 by treatment groups)

*** Groups ***	*** Baseline ***	*** Week 2 ***	*** Week 4 ***
Eucerin			
Mean ± SD	9.73±0.79	9.45±.87	8.42±.87
Median (range)	9(8–11)	9(8–10)	8(7–10)
Almond			
Mean ± SD	9.73±0.74	8.50±1.30	7.58±1.11
Median (range)	9(8–11)	9(6–10)	8(6–9)
Pumpkin			
Mean ± SD	9.88±0.62	7.45±.91	4.57±1.27
Median (range)	10(9–11)	7(6–9)	5(3–7)
Betamethasone			
Mean ± SD	9.76±1.67	7.87±1.33	7.31±1.23
Median (range)	10(7–11)	8(6–10)	8(6–9)
* P * -value	0.98	<0.001	<0.001

* DLQI: Dermatology life Quality Index

DLQI scores that measured at the beginning of the study in four groups had not significant difference. (*P*=0.98)

DLQI scores that measured in the day 14th and 28th day of the study in four groups had significant difference (*P*<0.001)

## Discussion

According to the findings of the study, the effectiveness in decreasing severity of chronic HE with betamethasone was more dominant than pumpkin group.

But it shoule be considred that after 4 wk of treatment, quality of life of pumpkin group improved significantly in comparison to the beta-methasone and other groups. Furthermore, pumpkin ointment is more effective in improvement of HECSI and DLQI scores compared to eucerin and almond ointment.

Chronic HE is the most common dermatological disorders. Its treatment options are abundant with limited efficacy (2). Current existing therapies are inadequate. Complementary and traditional medicines can be used as other approaches with those therapies (18). Among patients with chronic diseases, medicinal herbs are widely used in chronic illnesses (19, 20).

Pumpkin *C. moschata* is a medicinal plant cited in popular textbooks of traditional medicine, such as Canon of Medicine (10th and 11th centuries) (21), traditional Chinese medicine, traditional Indian medicine and other countries (22).

In recent decades, many scientific investigations have been done on pumpkin components because of widespread use of pumpkin in medicine (13). β-carotene, fatty acids, moisturizing agents, fibers, vitamins and carbohydrates are the main chemical composition of pumpkin pulp (23, 24). Oral consumption of pumpkin pulp has shown the adjunctive effect on treatments of many skin disorders such as dermatitis (12, 25, 26).Cutaneous adverse effects of topical corticosteroids occur regularly with prolonged treatment. The most frequent adverse effects include atrophy, striae, rosacea, perioral dermatitis, acne, and purpura (27, 28).

Although HE is not life threatening it can have unpleasant effects on patient’s quality of life such as limiting daily activities or losing social relationships (29). Improvement in quality of life after 4 wk was significant in pumpkin ointment. Additionally, patients that used pumpkin ointment reported no side-effect. Therefore, pumpkin ointment could be recommended as a substitute ointment for corticosteroids or as an adjunct therapy for HE.

We did not observe any allergic reactions in the skin or any deterioration in the eczematous lesions. Many researches have focused on scientific evaluation of the characterization of pumpkin principal nutritional components and preparation as food or medicine functional components. Popularity of pumpkin in various systems of traditional medicine for several ailments such as anti-inflammatory, anti-diabetic, anti-hypertensive, anti-tumor, immunomodulatory, anti-bacterial, anti-hypercholesterolemia, intestinal anti- parasitic and hepato-protective focused the investigators’ attention on this plant (22,30). Pumpkin oil has been used in skin eczematous lesions in traditional medicine and it may reduce skin damage (22).

However, the present study is the first trial of pumpkin flesh oil in the treatment of chronic HE and we found no previous reports on the effect of pumpkin flesh oil on eczema. Therefore, it was not possible to make comparison of this study with others trials. Only in a study, the effect of pumpkin seed oil on acute and chronic cutaneous inflammation induced dermatitis in mice was evaluated (31). In acute model, the ear swelling was measured at 1 and 4 h after xylene- and 12-O-tetradecanoylphorbol acetate-induced ear edema. The effect of treatments was measured each 24 h post-challenge with oxazolone-induced dermatitis for 4 d in mice in the chronic model. Topical pumpkin seed oil alters inflammatory response and resolves a persistent inflammatory lesion like dexamethasone without cutaneous alterations caused by topical corticosteroids (31). Additionally, this clinical trial was conducted in human model and to evaluate the severity of HE and quality of life, we used HECSI and DLQI questionnaire, respectively. Flavonoids and carotenoids are the components of pumpkin that have antimicrobial, anti-inflammatory, anti-histaminic, anti-oxidant and anti-cancer effects (30, 32–34). Hand dermatitis is a skin problem with skin barrier impairment, dryness is the cause of this problem, and moisturizing is the most important step in chronic HE management (35–37). Furthermore, the fruit moisturizing component is another important pumpkin’s property according to Persian scientists’ point of view, to recommend pumpkin ointment for chronic HE, the same as conventional physicians (24, 26, 38, 39).

The first limitation in this trial was the small sample size due to the lack of previous studies about effect of pumpkin on chronic HE. Other limitation was the lack of using patch test. It was not performed because previous studies that used edible pumpkin, had not mentioned any allergic or eczematous adverse effects.

## Conclusion

Pumpkin ointment, improved quality of life significantly. It could not reduce the severity of chronic HE as betamethasone, as demonstrated by assessing HESCI. Our study recommends pumpkin ointment as an adjuvant therapy with betamethasone ointment in order to reduce the amount of topical corticosteroids consumption.

## Ethical considerations

Ethical issues (Including plagiarism, informed consent, misconduct, data fabrication and /or falsification, double publication and /or submission, redundancy, etc.) have been completely observed by the authors.
